# Suicide Contagion: A Systematic Review of Definitions and Research Utility

**DOI:** 10.1371/journal.pone.0108724

**Published:** 2014-09-26

**Authors:** Qijin Cheng, Hong Li, Vincent Silenzio, Eric D. Caine

**Affiliations:** 1 HKJC Centre for Suicide Research and Prevention, The University of Hong Kong, Hong Kong SAR, and Department of Psychiatry, University of Rochester Medical Center, Rochester, New York, United States of America; 2 School of Management, University of Chinese Academy of Sciences, Beijing, China, and Department of Psychiatry, University of Rochester Medical Center, Rochester, New York, United States of America; 3 Injury Control Research Center for Suicide Prevention and Department of Psychiatry, University of Rochester Medical Center, Rochester, New York, United States of America; 4 VA Center of Excellence for Suicide Prevention, Canandaigua, New York, United States of America; University of Vienna, Austria

## Abstract

**Objectives:**

Despite the common use of contagion to analogize the spread of suicide, there is a lack of rigorous assessment of the underlying concept or theory supporting the use of this term. The present study aims to examine the varied definitions and potential utility of the term *contagion* in suicide-related research.

**Methods:**

100 initial records and 240 reference records in English were identified as relevant with our research objectives, through systematic literature screening. We then conducted narrative syntheses of various definitions and assessed their potential value for generating new research.

**Results:**

20.3% of the 340 records used *contagion* as equivalent to clustering (*contagion-as-cluster*); 68.5% used it to refer to various, often related mechanisms underlying the clustering phenomenon (*contagion-as-mechanism*); and 11.2% without clear definition. Under the category of *contagion-as-mechanism*, four mechanisms have been proposed to explain how suicide clusters occurred: transmission (*contagion-as-transmission*), imitation (*contagion-as-imitation*), contextual influence (*contagion-as-context*), and affiliation (*contagion-as-affiliation*). *Contagion-as-cluster* both confounds and constrains inquiry into suicide clustering by blending proposed mechanism with the phenomenon to be studied. *Contagion-as-transmission* is, in essence, a double or internally redundant metaphor. *Contagion-as-affiliation* and *contagion-as-context* involve mechanisms that are common mechanisms that often occur independently of apparent contagion, or may serve as a facilitating background. When used indiscriminately, these terms may create research blind spots. *Contagion-as-imitation* combines perspectives from psychology, sociology, and public health research and provides the greatest heuristic utility for examining whether and how suicide and suicidal behaviors may spread among persons at both individual and population levels.

**Conclusion:**

Clarifying the concept of “suicide contagion” is an essential step for more thoroughly investigating its mechanisms. Developing a clearer understanding of the apparent spread of suicide-promoting influences can, in turn, offer insights necessary to build the scientific foundation for prevention and intervention strategies that can be applied at both individual and community levels.

## Introduction

Despite the increasingly common invocation of the term “contagion” (hereafter denoted as *contagion* when considering its specific meaning) to analogize the spread of suicidal thoughts, behaviors, and deaths [1], [2], [3], there has been scant effort to rigorously assess the underlying concept or theory supporting the use of this term, or appraise its practical utility. In a related vein, authors have invoked an “epidemic” image for the apparent rapid increase in death rates resulting from such contagion. Undoubtedly, there have been review papers regarding suicide clusters [4], [5], the relationships between suicide and the traditional media [6], [7], [8], as well as the Internet [9], and even social contagion of non-suicidal self-injury [10]. However, as far as we know, there has been no systematic review specifically examining the concept and research methods of suicide contagion, and little standardization in the use of the term itself.

According to the Oxford English Dictionary and the Merriam-Webster Dictionary, the word *contagion* was formed in Late Middle English from Latin *contagio(n-)*, which is a combination of *con*- “together with” and the base of *tangere* “to touch.” It was, as stated by Oxford Dictionary, originally used to denote contagious disease and now is often used in two ways – referring to 1) a disease spread by the close contact of one person to another; and 2) the spread of a harmful idea or practice. The announcement of disease contagion often is associated with fear and panic among the public, given the concern that a disease is communicable (or transmissible) and spreading. Accordingly, the public would expect that efforts would be initiated to contain or restrict the disease’s dissemination through isolating or eliminating all sources of spread. However, as shown in Merriam-Webster Dictionary, *contagion* in contemporary English has become looser in its use and can also refer to rapid communication of an influence, which is not necessarily harmful, such as a doctrine or emotional state.

When *contagion* is used in the suicide research and prevention literatures, how do authors interpret this term? Do they use it in a consistent fashion? Specifically, we aim to clarify the definition and theoretical utility of *contagion* for promoting suicide-related research.

## Methods

**Figure 1:**
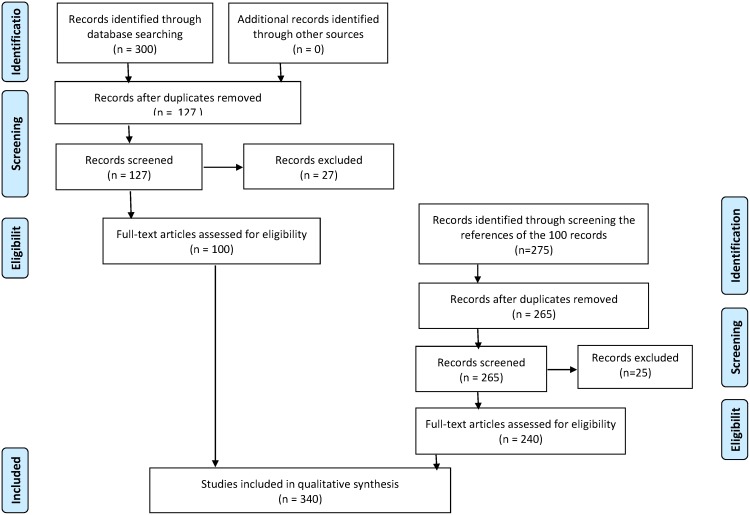
Flow diagram of literature search and review process.

We started our literature review with a broad scope to obtain a comprehensive picture of the academic use of *contagion.* As illustrated in Figure 1 and shown in Checklist S1, following the PRIMSA procedures [11], a literature search was conducted in three online databases: PubMed, Web of Science, and Social Sciences abstracts and full-text (via EBSCO) on April 4, 2013. Our literature screening included two steps, the first step is shown as the flow on the left side of Figure 1 and the second on the right. During the first step, Keywords “[Suicid] AND [Contag]” were searched in the titles and abstracts of all the archives, from earliest time period to the present, in the databases. One hundred twenty-seven records were identified after excluding duplications and another 27 records were further excluded (leaving 100 “initial records” for initial screening) because they did not meet one or more of our three entry criteria: 1) must be academic publications, including empirical studies, reviews, commentaries, and discussion pieces published in academic journals, books, and conference proceedings; 2) must explicitly use *contagion* or *contagious*; and 3) have full-text available in English. When we could not extract full-texts directly from the databases, we would contact corresponding authors to request help. Two authors kindly supplied full texts of their papers for the review.

Then we moved to the second step by reviewing all the 100 initial citations to identify those matching all the following criteria: 1) they have to be academic publications; 2) their content has to be cited by the initial records for defining or explaining *contagion*; and 3) have full-text available in English. From these we identified 240 references (hereafter referred to as “reference records”), excluding those included among the initial papers. Altogether, 340 records were included in our review.

We used content analysis to identify the definitions and theoretical bases of contagion in each record. The content analysis was guided by two specific objectives: 1) to summarize the definition of *contagion* and the authors’ opinion of whether suicide is contagious; and 2) to summarize theoretical bases and underlying dynamics of contagion. The first author reviewed all the 340 records and developed an initial coding protocol. When there was any uncertainty, the first author would discuss with the third and fourth authors to refine the coding protocol until agreement was achieved. Then, 34 records were randomly selected from the total records (10%) and coded by the first and second authors independently, using the refined coding protocol. Inter-rater reliability measured by the Kappa statistic was 0.85 (*p*<0.001), indicative of outstanding agreement. Subsequently, the first author used the refined coding protocol to re-code all the 340 records.

We considered performing a meta-analysis. However, we found a notable absence of analytic studies among the reviewed records. Moreover, the objectives, sampling methods, measurement of *contagion*, and statistical analytic methods of the records were very heterogeneous, precluding quantitative meta-analytic methods. Nevertheless, we conducted qualitative syntheses and assessed the utilities of different definitions of *contagion* using the following guidelines: 1) the definition should specific and differentiated from other concepts; 2) it should be consistent with conventional definition of contagion in medicine and public health; and 3) the theoretical basis of the definition should be logically robust and empirically testable, adding new perspectives for studying suicide, and not simply accepted as self-evident (e.g., the presence of a cluster indicates that something is contagious).

## Results

The 340 combined initial and reference records were published between 1960 and 2013. Details of our classifications of the 340 records are shown in Table 1. In terms of record types, most were original studies (68.8%). In terms of research topic, the majority of these records focused on suicidality (74.7%). The remainders were concerned with self-harm, risk behaviors or stress/depression/mood, which were cited by initial records to define or explain *contagion* in suicide. Of the 340 records, half did not specify the age groups they were studying, whereas the other half precisely focused on adolescents and young adults.

**Table 1:** Classification of the reviewed records.

			Frequency	% (N = 340)
**Record type**	Original studies		234	68.8
	Review papers		51	15.0
	Letters/editorials/commentaries		22	6.5
	Book chapters/conferenceproceedings/practicerecommendations		15	4.4
	Replicative studies		10	2.9
	Case reports		8	2.4
**Research topic**	Suicidality (e.g. suicides, suicide attempts,suicidal ideation, suicidemethods, and suicide bombing)		254	74.7
	Risk behaviors (e.g. violence, smoking,alcohol consumption, risky sexualbehaviors, etc.)		53	15.6
	Self-harm/self-aggression/self -mutilation		20	5.9
	Stress/depression/mood		3	0.9
	More than one topic(e.g. suicide and self-harm, suicideand other risk behaviors, etc.)		10	2.9
**Age of study objects**	No specify		173	50.6
	Adolescents, children, or/and young adults		167	49.4
**Definition of contagion**	*Contagion-as-cluster*		69	20.3
	*Contagion-as-mechanism*	*Contagion-as-transmission*	30	8.8
		*Contagion-as-imitation*	212	62.4
		*Contagion-as-context*	10	2.9
		*Contagion-as-affiliation*	3	0.9
		Mixed	36	10.6
		No clear description of mechanism	49	14.4
	No clear definition		38	11.2

As shown in Table 1, use of *contagion* among the reviewed records can be categorized in two groups: 69 records used *contagion* as the equivalent of clustering phenomenon (*contagion-as-cluster*, hereafter) [e.g. 12,13,14,15,16], while 233 records used *contagion* to refer to various, often related mechanisms underlying clustering phenomena (*contagion-as-mechanism*, hereafter). Further confounding the discussion, 38 records published papers used *contagion* without clear definition [e.g. 17,18], conflating possible interpretations. Below we focus on the three different manners to review the utility, theoretical bases, and research methods in each category.

### Contagion-as-cluster

*Contagion-as-cluster* records borrowed the term *contagion* from epidemiology research to label acute occurrence of similar behaviors among a group of people, who often share some sort of proximity. Some records also addressed the phenomenon as “suicide cluster” or “suicide epidemics” [19], [20]. There was little specification or agreement regarding the time interval required to qualify as “acute,” nor clarity regarding the social or spatial distance necessary to be considered “proximate.” Among the reviewed records, the range of time period varied from within 24 hours [21], a few days [22], to one year [23]. The range of distance varies from the same institution [15], [22] to the same region [23], [24]. Without either *a priori* standardization or empirical demonstration of metrics for time, space, or social connection, the process for defining connection or contact becomes potentially unreliable. As well, we found no consensus or common approach to establishing the degree of similarity among suicidal behaviors to quality as a “cluster.” While a few studies grouped suicide deaths by using the same method [17], [23], many lumped suicides using various methods, or combined suicide deaths and suicide attempts.

The invocation of *contagion* in these records appears to have provided a meaningful analogy for the authors to use to describe the phenomenon and underscore their high degree of concern. However, unlike “clustering,” which describes proximity in time, spatial arrangement, or both, *contagion* implicates some type of “contact” mechanism though which a disease is spread. Guided by this presumption, authors who use clustering to infer contagion point to proximity as their evidence; in essence, it becomes a circular argument (i.e., closely occurring events, however defined, must be related and thus there must be *contagion*). A few studies argued that a suicide clustering effect could be explained by the poor management of an institution [25] or special economic environment [26], rather than individual-level social contacts. Therefore, using *contagion* as equivalent to *cluster* is not robust heuristically – that is, it does not open doors that could lead to new research – and inadvertently, it may have constrained research by not encouraging investigators to more deeply explore the phenomenon.

### Contagion-as-mechanism

Under the category of *contagion-as-mechanism*, four different but related mechanisms have been proposed to explain how suicide clusters occur, including: transmission (*contagion-as-transmission*), imitation (*contagion-as-imitation*), contextual influence (*contagion-as-context*), and affiliation (*contagion-as-affiliation*; also known as homophily or assortative relationships).

#### Contagion-as-transmission

*Contagion-as-transmission* proposes that suicide or suicide-related components may be transmitted within a specific community or institution and result in clustering phenomenon [e.g. 5,27,28,29,30]. In many respects, it is related to contagion-as-cluster: While cluster infers disease spread based upon proximity, transmission makes explicit the key concept inherent in the term *contagion*, specifically the notion of direct person-to-person disease spread. Johansson and colleagues wrote: “suicide cluster formation can be described metaphorically as a contagious ‘disease.’ When susceptible individuals with this ‘disease’ come together, they might super-infect one another, resulting in a suicide cluster” [31].

When describing what might be transmitted person-to-person, authors have variously proposed: knowledge regarding specific lethal methods [30], impulsive-aggressive behaviors [32], stress in response to personal trauma [28], or simply, the willingness to perform suicidal behaviors [33]. *Transmission*, as used by these authors, ultimately reflects cognitive processes rather than a tangible element, such as a virus or bacterium. Thus, the term remains as a metaphor, which in turn leads us to conclude that *contagion-as-transmission* is a double-metaphor that adds little to “cluster” and nothing useful for stimulating the development a framework for future research.

#### Contagion-as-imitation

*Contagion-as-imitation* has been proposed as a stimulus-response process to explain suicide clusters, where interpersonal, group, and mass media communications serve as avenues for human contact or communication. Unlike *transmission*, *imitation* is drawn from psychology research and is more directly relevant with studies of human subjects.

Concern about imitative suicides can be traced to the 1770s when some readers of *The Sorrows of Young Werther* killed themselves in the same manner as Werther did [34]. Tarde, who wrote about imitation shortly before Durkheim [35], was a major proponent of *contagion-as-imitation*. However, Durkheim largely rejected Tarde’s views in his classic 1897 book, *Suicide,* even as he acknowledged that *imitative suicide* can occur [36]. He focused instead on the powerful effects of social integration and regulation in the modern era [37].

Sociologists after Durkheim did not pursue *contagion-as-imitation* as a mechanism for many decades [37]. Wheeler, a psychologist and major contributor to the reviewed records from the 1960s, proposed a theoretical concept of behavioral contagion [38]. In his definition, contagion occurs only under strictly defined circumstances. Specifically, Person X, who is prone to Behavior N (B_N_) but has not executed B_N_ due to powerful internal constraints, finally acts after being exposed to another person who performed B_N_ without punishment. Thus, Wheeler’s definition requires both a vulnerable person (i.e., one prone to action but inhibited) and another person who has carried out the act.

Wheeler clarified the concept of behavioral contagion by comparing it with social pressures and conformity, social facilitation, and imitation. In Wheeler’s opinion, the differences between contagion, social pressures/conformity, and social facilitation derived from the expectation that a person’s internal constraints were overcome by observing the behaviors of others. He argued that the lowering of the avoidance gradient in an approach-avoidance conflict was essential to the occurrence of contagion. Meanwhile, he cited a psychological dictionary to define imitation as “action that copies the action of another more or less exactly, with or without intent to copy” [39]. Wheeler, therefore, asserted that imitation is a generic term subsuming behavioral contagion, social conformity and pressure, and social facilitation. Most important for our review, the paradigm he proposed fostered experimental laboratory approaches to explore his theoretical argument.

However, Wheeler’s psychological perspective was not widely cited by later researchers. Criticizing Durkheim’s assertions and writing about *contagion-as-imitation* from a sociology perspective, David Phillips and others conducted a series of studies during the 1970s–1980s relating to suicide contagion [34], [40], [41], [42], asserting that “suicide, and other forms of violence, are contagious” [34]. Phillips’ research compared suicide rates in a society before and after some highly publicized suicides, work that was influenced by contemporary studies of population-level mass media effects [43]. Yet his notion of the Werther Effect as an example of *contagion* was roughly equivalent to what Wheeler had defined as *imitation*. Phillips took advantage of *contagion* as a medical term, and in turn proposed a group of hypotheses (Table 2) that broadened the investigation of suicide contagion to both individual and population levels [44]. As a result, Phillips’ studies were published in medical journals as well as sociology journals, and attracted broad academic attention. Not surprisingly, these studies are among the most frequently cited in our reviewed records and have essentially shaped the research agenda of *contagion-as-imitation* until present.

**Table 2:** From biological contagion to suicide contagion- concepts, utility, and implications, adapted from D. Phillips.

Analogy frombiologicalcontagion	Meanings in biologicalcontagion (Phillips, 1980)	Potential utility in suicideresearch (Phillips, 1980)	Findings in suicideresearch	Remained questions
Incubation period	The time delay between aperson being infected with amicroorganism and theappearance of symptoms	The time delay betweenfront-page publication ofsuicide news and increasingincidence of suicides.	Varies from threedays to one month	Not clear what factors influence the different findings of delay
		• Any relationship betweenthe nature of the front pagenews and the duration ofincubation period?		
		• Any relationship betweenthe susceptible people’scharacteristics and theduration of incubation period?		
Immunization	Sometimes people can be immunized against amicroorganism by beingexposed to weakened strainsof that microorganism	• Can frequent exposure tominor suicide stories buildup a person’s resistance tothe idea of suicide and thenimmune him against suicide?	NA	Phillips’ questions remain.
		• Potential equivalent ofbiological immunization isboredom or indifference towhat once would have been avirulent, powerful idea.		
Specific vs diffuse contagion	Specific contagion means aspecific connection betweena particular microorganismand a particular disease.Diffuse contagion means abroad connection betweena particular stimuli and a groupof correlated or complementaryresponses.	• Specific contagion: Aparticular type of suicidestories triggers one type ofsuicide and not another.	• Media reports ofan unusual suicidemethod (e.g. charcoalburning suicide,railway suicide)were associatedwith increase ofsuicide rates in thismethod.	Not clear what psychological or social mechanisms are underlying the association.
		• Diffuse contagion: thesame suicide stories maytrigger not only suicide butalso accidents, murders, orother violent behaviors.	• Phillips’ studiesexamined theassociation betweensuicide news andincrease in motorvehicle accident andairplane crash.	
Susceptibility to contagion	Persons in poor biologicalhealth are particularly easyto be contracted withbiological contagion.	Are persons who are inpoor cultural andpsychological health(e.g. anomic, have lowself-esteem and a pasthistory of failure) moresusceptible to suicidecontagion?	Adolescents are oftenfound morevulnerable	Did previous studies over sample adolescents but under-study other population groups?
Channels of infection	Some biological diseases arespread more efficientlythrough one mediumthan another.	Is newspaper more effectivein conveying suicidecontagion than television?Is word-of-mouth also aneffective channel oftransmitting suicide?	• More studies useddata from newspaperpublication andfound significantassociation betweenthe publication andfollowing increasein suicide rates.Findings fromtelevision and othermedia are lessconsistent.	Not clear whether and how multi-media channels can collectively influence suicide rates. Not clear how interpersonal, group, and mass media communication inter-act with each other on spreading suicide information.
			• Cohort studies ofpeer influence onadolescents andstudies of time-spacespecific clusters ofsuicide suggest thatinterpersonal orgroup communicationmay also influenceindividual’s suicidalideation or/andbehaviors.	
Quarantine	The spread of biologicalcontagion can be slowed orstopped through a quarantineof the infected individuals.	The less publicity given toan act like suicide, thesmaller the contagious effectof this act on others.	Decrease in publicitygiven to suicide newswas associated withdecrease in suiciderates in Austria. Somestudies evaluated theimplementation ofmedia guidelines ofsuicide news reportingand achieved mixedfindings.	Not clear whether the change of report quality or quantity (or both) can influence suicide rates.

Phillips noted that the *Werther Effect* occurs when suicides or violent behaviors were highly publicized (in essence rewarded), but he did not investigate in any depth what individual changes might occur to explain increased aggregate suicide rates. Nevertheless, his findings were consistent with the behavioral contagion theory that the publicity – notoriety – could serve to overcome internal constraints that had inhibited the occurrence of suicidal behaviors. In effect, Phillips work was guided by the same idea that Wheeler had proposed, that there are individuals who are susceptible (vulnerable) to highly publicized suicides that serve to overcome any vestiges of restraint. This argument was explicitly stated Gould’s work regarding suicide clusters [4], [45], [46], which in turn has been cited often by others.

However, we noted that few studies of *contagion-as-imitation* examined whether their subjects already were suicidal or definably vulnerable in some fashion before exposure to a publicized suicide, or whether the notoriety of these public deaths served as a “positive” stimulus for action. Among studies of *contagion-as-imitation*, there has been little consistency regarding the characteristics of the models (i.e., incident suicides) and the observers (i.e., exposed populations or individuals) that should be assessed, and what outcomes should be defined *a priori* as evidence indicative of a contagion effect. Does dissemination of a specific type of suicide trigger only suicides using the same methods? Or can it trigger suicides using diverse methods, as well as so-called accidents, murders, or other violent behaviors? In Phillips’ terms (see details in Table 2), is *contagion* from a suicide specific to future suicides or does it more generally stimulate other forms of risk-related premature death?

Phillips use of *contagion* as a metaphor served to transform the discussion in the field; however had he gone into greater depth to broadly analogize suicide an infectious disease, he would have had to consider concepts such as basic reproduction or infection rates and disease recovery, two important domains in infectious disease studies. [47]. Perhaps he regarded death as the sole outcome, excluding the possibility of recovery. However, infectious diseases are not universally fatal, a quality captured in the case fatality rate or percent. Similarly, suicide attempts differ dramatically in their fatality rates, depending upon the sex and age of the attempter and the method chosen [48]. The infectious disease analogy has much to offer, especially as investigators embark on studies of: (1) personal and impersonal communication networks through modern social media, (2) affect spread, and (3) the possible interplay of suicide-prevention and suicide-promoting media outlets. It would be valuable to examine whether individuals can recover from or “re-contract” influential communication threads involving suicidal thoughts or behaviors. In addition, the absence in Phillips’ framework of a basic reproduction rate, a metric indicating the extent to which an infectious disease can spread out through a population, suggests that he may have considered suicide contagion as one-time, one-way propagation, instead of continuous, variable, reciprocal, or recurring in transmission.

In summary, we consider *contagion-as-imitation* as a potentially dynamic set of processes that may include both individuals and populations. It provides, potentially, a framework for considering diverse perspectives – sociological and psychological, and medical and public health. Researchers such as Wheeler and Phillips have proposed structured and empirically testable hypotheses when defining *contagion-as-imitation*.

Therefore, *contagion-as-imitation* meets our three criteria for assessing definition quality, and shows potential as a theoretically useful concept that can stimulate new empirical studies. Of note, many authors did not use the more precise, research-oriented definition of *contagion-as-imitation* as carefully as Wheeler or Phillips. The latter were parsimonious and used the term to generate new inquires; others too often wrote as if everyone shared common understanding at a time when we know little about what underpins the apparent spread or diffusion of fatal self-destructive events.

#### Contagion-as-context

Only a small number of papers exclusively adopted *contagion-as-context* mechanism and most of them were based on ideas pertaining to peer influence and group norms [49], [50], [51], [52]. One version of this mechanism, summarized by Gould and her colleagues, is that a perceived change of group norms, as represented by a suicide, may serve to lower the threshold at which suicidal behavior is seen as an acceptable response to significant life stresses [4]. Another, proposed by Stack, is that publicized suicide stories may serve an agenda-setting function by shaping public opinions. These may be perceived by some as compelling, and in turn lead to imitative behaviors among vulnerable persons [53], [54]. Stack developed his explanation based upon behavior decision theory [55], reference group theory [56], and proposals regarding anomie [57].

*Contagion-as-context* appears to have been used by some authors in a fashion similar to *contagion-as-imitation*. However, we understand context and imitation as different, potentially complementary processes. *Contagion-as-context* refers to broadly acting processes, ones that can change community norms or sensitivities. Many may be exposed but only a few are vulnerable, based on other life experiences or personal factors (e.g., a specific psychopathology). The susceptible person need not have been exposed directly to the incident suicide or heard of the death. Instead, living in the same community might serve as a necessary factor. Given that collective norms are relatively stable and difficult to modify, *contagion-as-context* likely takes effect slowly compared with *contagion-as-imitation*, perhaps requiring significantly notable social events to instigate effects. In a related fashion, undoing or overcoming these influences may require sustained efforts over an extended duration to recalibrate social norms that serve to constrain self-harming and violent behaviors.

Given this perspective, the spreading of certain suicidal behaviors following contextual changes should be distinguished from contagion. When used in the public health and medicine literature, *contagion* has been either implicitly or explicitly linked with relatively rapid (acute); for example, through spread from person-to-person contact or through surface contacts (e.g., Ebola-laden fluids or airborne-carried flu virus resting on common surfaces). This type of relatively direct proximity transmission is not the basis for *contagion-as-context*. At the same time, we see great potential utility exploring how contextual influences potentially may moderate *contagion-as-imitation*.

#### Contagion-as-affiliation

Joiner described “assortative relating” [58], while Prinstein and colleagues [59], [60] used “homophily,” to denote clustering phenomena where people affiliate or assort with others possessing similar characteristics or sharing like-minded attitudes [5], [58], [59], [61]. We would prefer “affiliation,” a common term that relates both to making ties and being a member of a group of like-minded individuals. Some authors have described *affiliation* as a competing mechanism with *contagion* to explain suicide clusters [5], [58], [62]; e.g., Pristein and colleagues referred to it as a mechanism underlying contagion [59], [60] – another example of the literature’s inconsistency. *Affiliation, homophily, and assortative relating* all pertain to forming close ties with persons sharing common interests, values, and behaviors – independent from suicidal behaviors. Affiliation may be considered a more specific version of *context*, one that reflects relationships that long preceded the occurrence of suicidal behaviors. Such close ties may facilitate the spread of adverse behaviors, depending on the characteristics of the group, or they may serve to protect group members from environmental stresses and social strains, as the US Centers for Disease Control and Prevention has described by discussing “connectedness” as a key to suicide prevention [63].

Thus, affiliation would seem to be complementary to imitation, consistent with recent developments in social network research [64], [65]. There are no clear definitions regarding which factors or characteristics should be checked to measure the strength of affiliation, or what constitutes a group that is protective versus a group that increases the probability of adverse outcomes. Without being precise in their use of terms, suicide researchers may further head in a self-evident loop when seeking to examine *affiliation (assortative relationships; homophily)*.

## Discussion

The remarkable lack of clarity and inconsistent definition of “contagion,” as evident in our systematic review, has two major effects. It allows authors to cite one another to support their arguments without having to delve into the differences that separate them. One might say that it now serves as ‘accepted knowledge,’ without appreciating its assumptions and the profound gaps in understanding that are evident on closer scrutiny. Uncritically using *contagion* to paper over these gaps impedes attempts such as ours to succinctly summarize and synthesize the literature, and most importantly, to establish a solid foundation for future research.

Through review various uses of *contagion*, we assessed the potential heuristic utility of *contagion-as-imitation* as greater than *contagion-as-cluster*, *contagion-as-transmission*, *contagion-as-context*, and *contagion-as-affiliation*. *Contagion-as-cluster* is circular; clustering itself is the manifestation that prompts consideration of spreading influences. *Contagion-as-transmission* is, in essence, a double or internally redundant metaphor, as we noted previously. *Affiliation* and *context* (frequently examined in social network research) refer to influences that can affect health and illness, and occur independently from *contagion*, even as they may serve as a facilitating background (moderator). Phillip’s framework and Wheeler’s definition of behavioral contagion have provided structured and empirically testable hypotheses suitable to exploring *contagion-as-imitation*, although the framework can be further refined. Even though *contagion-as-imitation* has been used by some scholars without precise definition, the term itself potentially provides the greatest utility for examining whether and how suicide and suicidal behaviors may spread among persons at both individual and population levels.

We would like to stress that the present study has been concerned with the use or misuse of the term “contagion.” We have not, however, examined how knowledge or commentary regarding suicide may affect individuals and populations, nor have we discussed whether the possible spread of both adverse and protective influences reflects common mechanisms. We are aware that *contagion* has been used by some social science researchers to analogize rapid communication of neutral or positive influences, such as doctrines or emotional states. However, our review did not identify any suicide-related publications that have used *contagion* to refer to the spread of suicide preventive information or activities. Following our review, we also would like to suggest that if one intends to use a well-developed analogy, it should be used precisely – *contagion* is not used for positive attributes in the public health arena.

We must remain alert to possible biases in this literature. Most studies were conducted in the U.S., with the primary “vector” of contagion was institutional communication or mass media. In terms of mass media, newspapers and television films were studied much more than other media, such as television news [42], [66], [67], country music, book [68], and the Internet [68]. One must be aware of the emerging media landscape during this time of rapidly changing modes of communication. In addition, the studies we reviewed most often involved adolescents and young adults, and some suggested that youth are more susceptible to the clustering or the *Werther Effect* when compared to the general population [5], [12], [69], [70], [71]. The index suicides in those studies also were younger or the focus for youth culture, particular younger celebrities or pop idols. Indeed, among the reviewed records, there was no study that included the suicide of an elder as the stimulus case. We remain uncertain whether the apparent greater vulnerability of young people to *contagion-as-imitation* reflects a fundamental difference among persons from different age strata or a bias reflecting the greater attention paid to suicides among youth and young adults [72]. As well, because our content analysis was conducted primarily by one coder, we are sensitive to concerns about coder bias. We made certain to include all authors in different aspects of the coding process to minimize personal bias, and the recoding of 10% of our records by the second author revealed excellent inter-rater agreement.

## Conclusion

The present paper considers the myriad uses of *contagion* in the suicide research literature. When applied as a descriptive metaphor, to say that the apparent spread of suicidal behaviors among persons or populations is a “contagion,” its use has illustrative and frightening power. We asked ourselves in this paper – and in turn, the larger field – whether such a broadly applied term serves to generate new research and understanding. Many authors have adopted the term as an analogy, with complex implications regarding shared characteristics and presumed mechanisms of spread that have scant scientific support. Once adopted, *contagion* has been oft-repeated without critical scrutiny of its uncertain meanings, or implied mechanisms, and the presumed clarity of what authors have intended to say has only served to mask or mute rigorous inquiry.

Based on current data and appreciating the substantial limitations in published studies that we have described, it is problematic to draw any conclusion whether suicide truly is contagious – that is, passed from one person to another, either directly or indirectly. Undoubtedly, clusters are apparent and well described and population-level fluctuations have been demonstrated after key events. These do not prove contagion! Our review suggests that the concept of suicide contagion requires further investigation, and its use (along with terms such as *epidemic*) should be defined cautiously and thoughtfully.

For research and prevention purposes, *contagion-as-imitation* promotes the use of sociological and public health perspectives, as well as individually oriented psychological constructs, and appears to have greater heuristic utility than other uses of the term. It opens the door to network science and the burgeoning world of research involving social media. At the same time, understanding the nature of context and affiliation are essential, as these likely serve as powerful influences that shape both risk and protection: Integrating the effects of what may be slower-developing life circumstances (i.e., factors related to context, such as community and family, and personal affiliations) with faster-developing life events (e.g., exposures, personal conflicts) is a major challenge for understanding the forces that contribute to an individual’s unique vulnerabilities and resilience.

It is our hope that this review encourages researchers to explore competing and complementary mechanisms that may explain repeated observations of clustering and apparently rapid population fluctuations in suicide rates following highly publicized suicides. On a larger scale, such research may cast a light on larger scale poorly understood issues involving longer-term temporal variations in suicide rates within countries or regions, notable differences in rates within and between countries, and the apparent local preferences for selecting one method over another, even as both may be readily accessible. Such research has the potential to foster new prevention initiatives that may saves lives during ‘acute’ events while also offering avenues to broader public health programs that seek to profoundly transform the prevalence of suicide within and among nations.

## Supporting Information

Checklist S1 **Filled PRISMA Checklist.**
(DOCX)
